# Ten simple rules to cultivate transdisciplinary collaboration in data science

**DOI:** 10.1371/journal.pcbi.1008879

**Published:** 2021-05-13

**Authors:** Faryad Sahneh, Meghan A. Balk, Marina Kisley, Chi-kwan Chan, Mercury Fox, Brian Nord, Eric Lyons, Tyson Swetnam, Daniela Huppenkothen, Will Sutherland, Ramona L. Walls, Daven P. Quinn, Tonantzin Tarin, David LeBauer, David Ribes, Dunbar P. Birnie, Carol Lushbough, Eric Carr, Grey Nearing, Jeremy Fischer, Kevin Tyle, Luis Carrasco, Meagan Lang, Peter W. Rose, Richard R. Rushforth, Samapriya Roy, Thomas Matheson, Tina Lee, C. Titus Brown, Tracy K. Teal, Monica Papeș, Stephen Kobourov, Nirav Merchant

**Affiliations:** 1 Data Science Institute, University of Arizona, Tucson, Arizona, United States of America; 2 Computer Science Department, University of Arizona, Tucson, Arizona, United States of America; 3 BIO5 Institute, University of Arizona, Tucson, Arizona, United States of America; 4 National Museum of Natural History, Department of Paleontology, Washington, District of Columbia, United States of America; 5 Steward Observatory and Department of Astronomy, University of Arizona, Tucson, Arizona, United States of America; 6 CODATA Center of Excellence in Data for Society, Washington, District of Columbia, United States of America; 7 School of Information, University of Arizona, Tucson, Arizona, United States of America; 8 Native Nations Institute, University of Arizona, Tucson, Arizona, United States of America; 9 Center for Digital Society and Data Studies, University of Arizona, Tucson, Arizona, United States of America; 10 Fermi National Accelerator Laboratory, Batavia, Illinois, United States of America; 11 Kavli Institute for Cosmological Physics, University of Chicago, Chicago, Illinois, United States of America; 12 Department of Astronomy and Astrophysics, University of Chicago, Illinois, United States of America; 13 School of Plant Sciences, University of Arizona, Tucson, Arizona, United States of America; 14 CyVerse, University of Arizona, Tucson, Arizona, United States of America; 15 DIRAC Institute, Department of Astronomy, University of Washington, Seattle, Washington, United States of America; 16 eScience Institute, University of Washington, Seattle, Washington, United States of America; 17 Department of Human Centered Design and Engineering, University of Washington, Seattle, Washington, United States of America; 18 Department of Geoscience, University of Wisconsin–Madison, Madison, Wisconsin, United States of America; 19 Instituto de Ecología, Universidad Nacional Autónoma de México, Mexico City, Mexico; 20 College of Agriculture and Life Sciences, University of Arizona, Tucson, Arizona, United States of America; 21 Department of Materials Science and Engineering, Rutgers University, Piscataway, New Jersey, United States of America; 22 Biomedical Engineering Department, University of South Dakota, Sioux Falls, South Dakota, United States of America; 23 BioSNTR, Brookings, South Dakota, United States of America; 24 National Institute for Mathematical and Biological Synthesis, University of Tennessee, Knoxville, Tennessee, United States of America; 25 Google Research, Mountain View, California, United States of America; 26 Pervasive Technology Institute, Indiana University Bloomington, Bloomington, Indiana, United States of America; 27 JetStream Cloud, Indiana University Bloomington, Bloomington, Indiana, United States of America; 28 Atmospheric & Environmental Sciences, University at Albany, Albany, New York, United States of America; 29 National Center for Supercomputing Applications, University of Illinois at Urbana-Champaign, Urbana, Illinois, United States of America; 30 San Diego Supercomputer Center, University of California, San Diego, La Jolla, California, United States of America; 31 School of Informatics, Computing, and Cyber Systems, Northern Arizona University, Flagstaff, Arizona, United States of America; 32 Planet Labs, San Francisco, California, United States of America; 33 NSF’s National Optical-Infrared Astronomy Research Laboratory, Tucson, Arizona, United States of America; 34 Department of Population Health and Reproduction, University of California, Davis, Davis, California, United States of America; 35 Dryad, Durham, North Carolina, United States of America; 36 Ecology & Evolutionary Biology, University of Tennessee, Knoxville, Tennessee, United States of America; Carnegie Mellon University, UNITED STATES

## Introduction

Despite the tremendous advances in data-driven platforms, technologies, and analytical tools designed to ease collaboration between researchers and data scientists, very little attention has been devoted to understanding or developing the culture of collaboration—i.e., how interpersonal dynamics between research professionals drive collaboration and the institutional roles that sponsors, universities, and experts play in the coproduction of knowledge. “Collaboratory cultures” is a people-first structure in the research ecosystem and necessary to support the next wave of data-driven “transdisciplinary” research. Individuals who possess the skills to lead transdisciplinary projects and the savvy to negotiate collaboratory cultures will be the most effective at advancing their research agendas.

**Fig 1:**
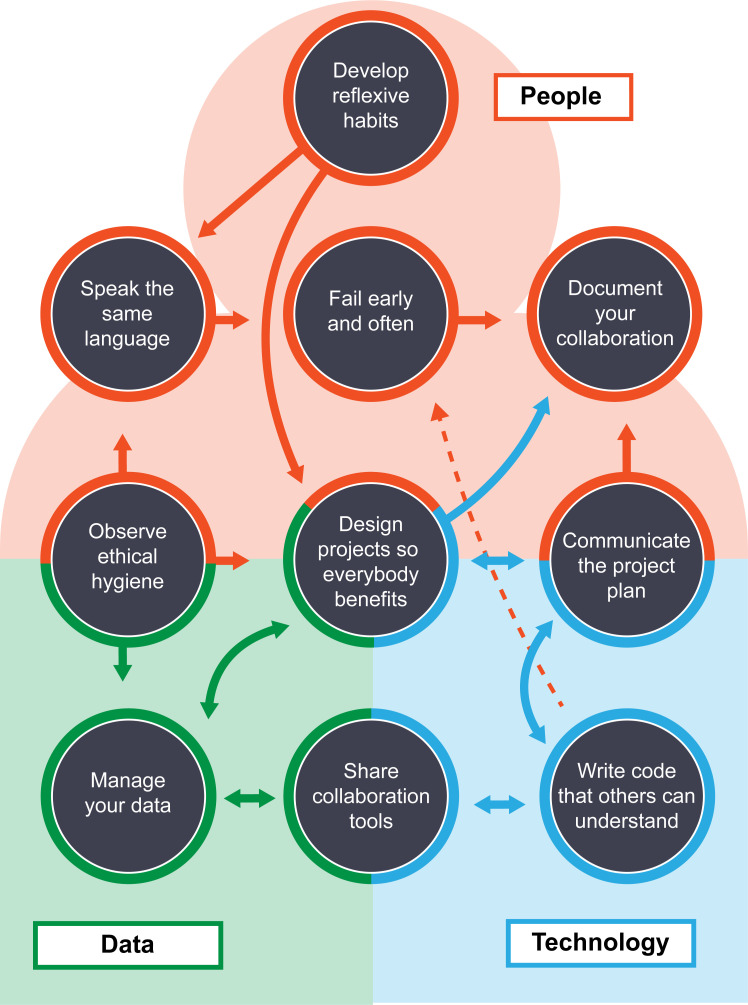
How the rules work together and intersect. There are multiple components in collaborations: person–person interactions, person–technology interactions, person–data interactions, and data–technology interactions. Synergy between these components results in a successful collaboration.

This article is the result of the 2019 Lemon Labs workshop [1], where the authors of this article shared their collective experiences on a wide range of data-driven science issues. This “visioning lab” event provided an open, inviting space for participants to share the challenges they face in their own collaborative projects (see S1 Text). Lessons learned were summarized and developed into the following interconnected (see Fig 1) Ten Simple Rules within a “collaboratory cultures” framework. While many Ten Simple Rules have been written about general collaboration, data sciences collaboration, statisticians’ collaborations, and leveraging big data [2–7], we emphasize the “nontechnical” criteria that are necessary to promote effective collaborations, accelerate discovery, facilitate new partnerships, and develop the role of individuals within transdisciplinary [8] research projects—projects that combine disciplines in a nontraditional way, resulting in the development of novel frameworks, concepts, and methodologies to address scientific problems.

We provide guidelines for investigators who have the need for and the interest in a new collaboration but do not know how to get started, as well as for those already starting collaborations but do not know how to structure newly formed teams in a way that will produce the desired outcome. Although these guidelines describe research collaboration between the data sciences and the disciplinary sciences, their application is not limited to the academic sector. These principles work for both in-person and remote collaborations and provide a pathway to build upon the “collaboratory” concept as it was first envisioned by William Wulf [9], particularly in the utilization of communication technologies and digital collaboration tools to “support” collaboration, but not to “replace” the societal function of successful research teams.

Table 1 provides a glossary of key terms used in this article.

**Table 1:** Glossary of certain terms for the purpose of this article.

Term	Definition
Data Scientist	An expert in wrangling, manipulating, and analyzing data.
Disciplinary Researcher	Researcher with specific knowledge within a science field, such as Astronomy, Biology, Sociology, etc.
Collaboratory	A portmanteau of the words “collaboration” and “laboratory” [9,10], which encompasses the research capacities that are enabled by digital communications, big data tools, and multidisciplinary researchers; the open research environments that allow colleagues to share, access, and use digital resources and instruments freely [9]; and the coproduction of knowledge through multiple forms of communication, both formal and informal, and a shared agreement of values and rules [11].
Infrastructure	Underlying foundation to do work. Examples include lab manuals, platforms for sharing and analyzing data, etc.
Platforms	Software designed to help manage and disseminate data storage and analyses.
Standard Operating Procedure	Ground rules between team members which assigns roles, modes of communication and designates timelines.
Tools	Technologies for specific tasks (e.g., version control, communication).
Workflow	Pattern and order of operations for tasks that are part of a project.

## Rule 1: Develop reflexive habits

Reflexive practices involve the exploration of one’s own discipline through the lens of another discipline. Data scientists and researchers should engage in each other’s knowledge sharing and practitioner events.

In traditional academic settings, it is common for data scientists to be scattered across various departments. They participate in local hacking groups, meetups, Carpentries workshops [12], and public competitions, which serve to introduce the data sciences community to the problems and datasets within a specific scientific discipline. In order to better understand the interdisciplinary role that data scientists play within their own disciplines, it is important for researchers to gain knowledge and create relationships with the data sciences community by attending workshops, conferences, colloquia, hackathons, and meetups. Here, emphasis is on the collaboratory activity; the onus is not on one person to seek, attend, and learn, but on the research community as a whole to engage with each other. For example, events such as AstroHackWeek, Summer School in Statistics for Astronomers, Data Science for Social Good, Oceanhackweek, or the Google Earth Engine User Summits [13] provide opportunities for individuals to learn about innovations and advances in data technology within their own fields, while deepening connections with the data sciences community. These events may be especially beneficial for students and postdocs who are just starting their research careers and still finding their interdisciplinary niche.

Many organizations structure their events to facilitate new partnerships. But individuals may not know how to interpret the jargon, concepts, and tools unique to each discipline. Reflexivity can close these gaps by inviting new information into the local knowledge regime, opening a frame of reference for the potential of interdisciplinary relevance across departments, and introducing the jargon and concepts that are key to successful transdisciplinary efforts. The normative value of reflexivity is the permission it gives to researchers to spend the time and make the effort to find transdisciplinary spaces in their own research ecosystem. This creates space and time for collaborations to arise and succeed.

To adopt a reflexive approach, researchers and data scientists should schedule visits to each other’s institutions or departments, follow each other on social media, jointly apply for grants and submit proposals for interdisciplinary workshops, and attend less formal events, such as graduate seminars and department-sponsored socials. Conferences and in-house seminar series are a great networking opportunity for graduate students, so students should be encouraged to meet with speakers and invite them to department events. Even if the meeting does not result in a collaboration, the lab has learned new jargon and grown its data sciences network. These approaches facilitate collaborative cultures, as well as deepen and improve one’s disciplinary expertise.

## Rule 2: Communicate the project management plan early and often

Effective collaboration requires team leadership, personal motivation, and clearly defined project goals. Teams and team leaders should cocreate a project management plan with milestones and deadlines that lead to the desired output, assign roles and tasks according to the strengths and interests of each team member, and invest in the personal contribution of individual team members. In the academic environment in particular, where there is emphasis on training and development, members should be encouraged to participate in efforts that help them learn a new skill they are interested in.

Like all community efforts, scientific collaboration can suffer from a kind of “diffusion of responsibility” [14] that leaves the majority of work to be performed by a minority of the group after the initial planning period. This diffusion of responsibility can lead to negative experiences and have a chilling effect on future collaborations. Lemon Labs participants revealed that when collaboration stalled, progress required someone to take ownership of the project and reinvigorate the team (see S1 Text). To help ensure success in the design of the project management plan, team leaders should design Standard Operating Procedures (SOPs) (see Table 1) and communicate it to team members early on and disseminate updates. SOPs can strengthen the vision for collaboration by providing frameworks for thinking through the “what ifs” and “how tos” involved in the project lifecycle. They put the pieces of a project into context and preserve the integrity of the project’s goals over time.

The SOP should detail clear roles, responsibilities, authorship guidelines, and additional information, such as how to label project files (see Rule 6). A good SOP should incorporate team feedback in the design phase and map to project milestones with regular review and updates (see Rule 5). Components of a good SOP include:

- Defining the purpose of the collaboration;
- Assigning roles and responsibilities for all collaboratory members involved in the project lifecycle, including principal investigators and team leads;
- Outlining benchmarks of success (i.e., project milestones); and
- Defining collaboration tools and how they relate to the purpose of the project, such as communication platforms and meeting schedules (see Rule 6).

For a more thorough guidelines on writing a successful SOP, interested readers may refer to [15]. The SOP we designed to facilitate the collaborative writing of this article is available at [16].

## Rule 3: Speak the same language

Curiosity-driven dialogue constitutes the core value of collaboratory cultures because all science practitioners share a love of knowledge in the spirit of inquiry. Transdisciplinary teams should adopt an inclusive environment that encourages questions, fosters understanding of new concepts, and aids in vocabulary building.

The variety of backgrounds and competencies in complex knowledge production presents a further challenge to the cohesion of virtual teams. Simple tools, such as communication guidelines documented in the SOP (see Rule 2) and a glossary of terms, can help researchers and data scientists to understand each other across disciplines. But tools alone do not get the job done. Collaboration requires communication to share ideas and time to build knowledge. So, while team members might need to use jargon to explain concepts from their own disciplines, team leaders should factor in enough time for the thoughtful exploration of new concepts and the explanation of new terminology. These terms and concepts can then be reviewed iteratively until all jargon is well defined and understood across the team, and the concept itself becomes clear.

Cultivating an inclusive and welcoming environment is necessary to foster open communication. Cocreating shared values [17] and documenting them via *Codes of Conduct* [18–22] is a foundational building block in collaboratory cultures. This resource provides a valuable team-building exercise and helps to ensure that all team members—especially early-career researchers, graduate students, and members of marginalized or underrepresented communities—are effectively engaged in the collaboration, valued in their roles, and empowered to contribute their perspectives in equal measure. You can model welcoming leadership by incorporating a few simple guidelines:

- If you don’t understand, ask a question.
- If you do understand, answer the question.
- Respect and encourage a diversity of opinions, backgrounds, and experiences.
- Avoid jargon when a synonym can be used. When jargon is unavoidable, define terms.
- Be curious.
- Discuss conflicting ideas or approaches as they arise and call on the wisdom of the crowd to find creative resolutions.

These practices are only sustainable in a culture of acceptance and psychological safety, where leadership by example is crucial: practice active listening, create a space where questions are welcomed and celebrated, and frame “failures” as valuable learning tools in the solution process.

## Rule 4: Design the project so that everybody benefits

Transdisciplinary teams involve experts who possess a diversity of knowledge, experience, skills, interests, professions, capacity, seniority, and ambition. Project planning should incorporate the research agendas of all collaborators.

Most collaborations are based on the need for investigators to apply data science methods to produce a scientific outcome. Generally speaking, data scientists value “methods,” whereas disciplinary researchers value “results.” Often, the value of the collaboration is reduced to its scientific output without regard to the investment of the time and effort that is required to develop the novel data science solutions, which are frequently necessary to achieve the science community’s research goals. This lack of regard for the important contribution from the data sciences can lead to feelings of frustration, the perception that the role of the data scientist is merely a technical worker who does not directly contribute to scientific discovery, and the belief that the individual contribution of the data scientist is less important to the success of the collaborative effort (see S1 Text). Obviously, these negative experiences can interfere with the cultivation of a strong and productive collaboratory culture. Managing the contribution of each team member proactively allows all collaborators to invest equally in the project’s outcome, and to find motivation in their own contribution.

Include these considerations in the development of your project plan and elicit specific feedback from each team member to ensure that they have an appropriate role in the coproduction process:

- Create a flexible-by-design framework that can accommodate variable scope and unanticipated results. In other words, give room to both data scientists and disciplinary researchers to pursue what matters to them, while collaborating on the project;
- Specify the distinct contribution that each collaborator has to offer to their field;
- Identify inclusive objectives and/or outputs that allow each contributor to advance their own professional goals and research agendas;
- Account for differences in the fundamental approach to research between disciplines and practices, including methodology, experimental design, and analysis [23];
- Clarify that the results of collaborative research, including data science methods, will ultimately be evaluated by disciplinary experts;
- Do not assume that disciplinary contributions will contribute to the research portfolio of the data scientists, and vice versa; and
- Revise and improve your plan as you onboard new collaborators (see Rule 2).

## Rule 5: Fail early and often

Data sciences projects characteristically involve perceived failures in the short term, which often illustrate design weaknesses or oversights that can be corrected in time to deliver a successful result. Collaborative teams should fully embrace failure and learn to leverage these setbacks into opportunities for growth and success of their collaboration.

A major difference between traditional scientific studies and data science projects is the frequency of (mini) failure. For instance, a common experience shared by Lemon Labs attendees was code failure and the fear of committing code to a public platform. Do not wait until your code is perfect and flawless. Disclosing experimental successes and failures is a hallmark of scientific knowledge production. Consider how sharing your innovative efforts with others contributes to scientific knowledge, and the importance of understanding failures and mistakes, which is critical to process efficiency and improvement. Furthermore, to help encourage groups that may suffer from this fear—often early-career and underrepresented investigators—create a safe space for them to learn this practice and get into the habit of sharing their work.

Team members who feel disempowered to share their failures can stagnate the project. One tactic to overcome stagnation is to adopt agile strategies, such as conducting a periodic review of milestones, accomplishments, and challenges through the practice of “blameless retrospective.” This approach assumes that everyone did their best with the skills, tools, and time they were given, and encourages critical examination by workshopping what worked, what didn’t work, and what can be improved going forward. In this way, teams can effectively iterate failure into success.

People-first techniques like this also strengthen collaboratory cultures by allowing teammates to express questions and concerns about the project, explore new ideas together, and build trust through nonjudgmental discourse. It also provides a process-oriented framework, which can serve to ease interpersonal conflicts, especially when there is a perceived disparity in effort between team members.

## Rule 6: Share collaboration tools

All scientific knowledge is rooted in sharing—sharing discoveries, sharing ideas, sharing methods, and sharing data. Transdisciplinary collaboration should leverage the tools, skills, and resources that each member brings to the project by sharing them freely.

Collaborative tools drive cooperative research. They improve communication, accelerate discovery, facilitate relationships, broaden perspectives, and provide team members with access to new data-gathering methods and analytical devices. These tools should be organized prior to beginning the project. Instructions for access to project tools and descriptions of their purpose should be included in the SOP (see Rule 2).

The following collaborative tools facilitate communication and sharing among team members:

- *Collaborative documents* allow multiple users the ability to take notes, edit, and comment in real time.
- *Shared calendars* allow team members to schedule meetings, set deadlines, assign time-sensitive tasks, and track project milestones.
- *Instant messaging* allows real-time communication between members to ask and answer questions, and across teams make general announcements.
- *Video conferencing* allows multiple users to share video and screen content, regardless of location.
- *File sharing* provides real-time editing capability to multiple users, while supporting access control (i.e., “view only” versus “editing” access).
- *Data repositories* provide version control, multiple threads, and updates.
- *Project archives* provide permanent access to project assets and resources.
- *Reproducible and executable software configuration*, commonly referred to as *containerization* [24], bundles together software, configuration files, dependent versions, and data so that projects can be reproduced, regardless of future changes in operating systems and software versions.
- *Computational notebooks and tutorials* combine code, results, and descriptive text into a computational narrative [25].
- *Scientific discovery platforms* enable computational research and data science collaboration with big data assets.

## Rule 7: Manage your data like the collaboration depends on it

Because it does!

A shared set of standards and tools allows all project members to work together to organize data, collect metadata, improve data quality, and access data as needed. Collaborative teams should become proficient in data management best practices [26] and work together to create data management plans that support FAIR data principles (Findable, Accessible, Interoperable, and Reusable [27]).

In data sciences collaborations, the person who creates the data and interprets the results is often different from the person who analyzes the data. Every step of the data process should be saved, logged, and documented to allow researchers to access that data for analysis through the project lifecycle and to preserve context for future reproduction studies. The effort to make data FAIR—cleaning data, putting them into interoperable data formats, and publishing both code and data early and often in open source formats—is a necessary labor investment for the data sciences community, because it pays off downstream in the open access environment and supports scientific reproducibility. The immediate reward to the collaboratory is the on-demand access to data assets as soon as they have been processed according to FAIR standards. Indeed, such practices provide significant gains to individual researchers [28] by speeding up the revision process and giving their data broader reach across the open science community.

## Rule 8: Write code that others can use and reproduce

Data scientists can improve their skills by adopting some of the tools and habits of software engineers. They should prioritize the development of workable, readable, and executable code that can be reused by collaborators and researchers across a wide variety of disciplines [25].

Software engineers have decades of experience working in large teams to write complex code, organize workflows, and satisfy the competing demands of multiple stakeholders. Whether building analytics tools, cleaning data, or writing software, data scientists can benefit from observing some of the principles of software development. Always keep in mind that your code has value only if your collaborators and future users can continue to use and reuse your code. Training in software engineering practices offers the added benefit of delivering a set of valuable skills that are in high demand in the private sector.

Finally, strong code-writing skills foster ethical and responsible research outcomes, such as reproducibility. Reproducible research links convergent data science practices to scientific research and strengthens collaboratory cultures. These skills include deploying packages through online platforms, writing container “recipes,” using version control systems, developing workflows, efficiently storing data, and using data management systems. Table 2 lists a selection of good practices for creating sustainable, accessible codes. For a more thorough and technical best practices, interested readers may refer to [29].

**Table 2:** Suggested practices for creating sustainable, accessible codes.

Function	Suggested practices
Generic	• Provide a step-by-step user manual for tools whenever possible.
	• Provide high-level comments at the beginning of each file and throughout the code as needed.
	• Follow consistent naming convention across your codes.
	• Do version control.
Data curation	• Automate as many of the processes involved in data access, storage, and reformatting as possible.
	• Keep separate copies of the original (raw) data and the curated data.
Data analysis	• Research and employ common, successful analysis methods. Do not reinvent the wheel.
	• Map your method to the research questions you are trying to answer. Do not try to fit your method to an application.
	• Make a tutorial-style document that explains your analytical method in simple language.
Data visualization	• Use visual graphics to communicate the final result with collaborators.
	• Use high-quality formats to produce images.
	• Automate data visualization as much as possible.
	• Aim for users being only a single click away from reproducing everything.
Tool building	• If possible and where appropriate, build a Graphical User Interface (GUI) that allows your collaborators to tweak parameters and apply their expertise to parameter evaluation and exploratory analyses.
	• When appropriate, build add-on packages and libraries.

## Rule 9: Observe ethical hygiene

Ethical protocols are often dismissed as administrative functions that exist outside the pursuit of scientific knowledge. Researchers should stay current on best practices, observe ethical hygiene throughout the research lifecycle, and prioritize the ethical guidelines published by their research sponsors [30].

The arbitrary adoption of standards and application of rules creates barriers to collaboration across fields, innovation within fields, and the advancement of science throughout. Consistency in ethical hygiene is uniquely critical for data sciences projects because the methods, tools, or algorithm developed for one purpose are often repurposed within very different contexts or domains. This fungibility allows data scientists to move laterally across disciplines. However, when the application of data tools moves from one context, where ethical considerations were deemed irrelevant to the project outcome, to a different context that directly impacts human lives, the result can create harms throughout society. For example, facial recognition tools that were designed to improve image searching and social media functions have been adopted for use outside that context for racial profiling, cyberstalking, identity theft, deep fakes, and other applications that erode privacy and cause harm on a massive scale. Awareness of ethical concerns and best practices can help data scientists to design tools that are less prone to misuse.

Ethical hygiene also protects individuals from social, ethical, and legal liability in the workplace. These protections foster a welcoming environment by clearly stating the community’s values and rules of behavior, which in turn supports collaboratory cultures. In the absence of social norms, uniform rules, and universal regulations, the adherence to ethical protocols [18,31,32] can prevent individuals and institutions from engaging in harmful practices—intentional or otherwise. Rather than viewing ethical commitments as restrictive rules that hamper research, collaborators can reframe ethics as the shared expression of the research values that deliver the high-quality, reproducible outputs that can withstand critical scrutiny.

The National Science Foundation provides general guidance and discipline-specific ethical guidelines on its website [33] and also sponsors the Online Ethics Center for Engineering and Science (OEC; [34]), which offers extensive resources, training, events, curricula, case histories, best practices, and more to serve the science community. This coordinated effort reflects an increasing interest in accountability, responsibility, and ethics in scientific research. Materials found on the OEC website can be incorporated both in the classroom for student instruction and in the lab via Codes of Conduct included in a project SOP.

## Rule 10: Document your collaboration

The experiences, reflections, and evolving best practices that result from data science collaborations can benefit the entire research community by providing anecdotal evidence about what works. Transdisciplinary teams should regularly document their collaborative experiences, regardless of perceived successes or failures.

The *Fourth Paradigm*’s data-intensive science [35] framework is a collective, long-term enterprise. Project journaling normalizes the reflexive habits that support evidence-based best practices and reinforces collaboratory cultures, while providing teams with a running account of the significant events, divergent opinions, and decisions that directly impact project outcomes. It enhances project management by providing an outlet to reflect on experiences, celebrate successes, share lessons learned, and document change. This form of documentation translates into evidence-based guidelines that can be shared in the classroom, at conferences, during team meetings, and with future collaborators. It has the added benefit of developing expository writing skills, which are critical in effective science writing and interdisciplinary communication. And of course, the practice preserves important research for posterity, which helps cultural researchers trace revolutionary discoveries and interpret the impacts of science on society. The participants of Lemon Labs engaged in reflexive activities to help participants identify where collaborative projects have previously failed (see S1 Text). These activities throughout the workshop informed the rules included in this article.

## Conclusion

It has been over 30 years since William Wulf introduced his vision of interdisciplinary research without walls, which he termed the collaboratory—a portmanteau of the words “collaboration” and “laboratory” [9,10]—and more than a decade since Hey and colleagues described the emergence of data-driven research as a new scientific paradigm [35]. These Ten Simple Rules and their emphasis on collaboratory cultures provides a framework for sponsors, investigators, students, and other stakeholders to positively support each other in an increasingly virtual environment through the thoughtful selection of collaborative tools, best practices, and agile management techniques. Investigators are stretched thin, Big Science is designed to favor siloes and exclude underrepresented stakeholders, and research administration departments are not equipped to provide management services throughout the full project lifecycle. While the main takeaway for investigators is to better understand the cultural dynamics involved in transdisciplinary collaboration, it is also a call to action for research sponsors, who need to reconsider their investment in the costs related to complex knowledge production.

Finally, a note on reflexivity and best practices. This list of Ten Simple Rules was an outcome of extensive discussions held by Lemon Labs participants in 2019, in which we told our stories about project success and failure, workshopped workplace problems and solutions, and reached consensus about more effective ways of doing things. We have committed to adopting these rules within a reflexive framework over the next year by applying each rule and documenting implementations and outcomes. We will use our findings to deliver evidence of each rule’s effectiveness (or not) in specific collaborative projects.

## Supporting information

S1 Text Challenges. During the first 2 days of the Lemon Labs, participants shared about challenges that they commonly face in their collaboration. Here is a summary of “complaints” that participants shared during the meeting. We have also listed possible approaches to deal with them and provided reference to specific rules that would address them.
(PDF)
