# Supplementary material for: Ten simple rules to cultivate transdisciplinary collaboration in data science
Source: PLoS Comput Biol. 2021 May 13;17(5):e1008879. doi: 10.1371/journal.pcbi.1008879 (PMC8118297; doi:10.1371/journal.pcbi.1008879)
Supplement: S1 Text — During the first 2 days of the Lemon Labs, participants shared about challenges that they commonly face in their collaboration. Here is a summary of “complaints” that participants shared during the meeting. We have also listed possible approaches to deal with them and provided reference to specific rules that would address them. (PDF) [file pcbi.1008879.s001.pdf]

# Supporting information

**S1 Text. Challenges.** During the first two days of the Lemon Lab, participants shared about challenges that they commonly face in their collaboration. Below is a summary of "complaints" that participants shared during the meeting. We have also listed possible approaches to deal with them and provided reference to specific rules that would address them.

**Challenges pertaining to expectations**:

1. Data scientist interested in unnecessarily advanced algorithms
   - *Suggested approach to address them*: Set up expectations; Make sure questions satisfy all parties
   - *Relevant rules*: 2,4
2. Disciplinary researcher does not value method novelty and only cares about final result.
   - *Suggested approach to address them*: Set up expectations; Make sure questions satisfy all parties
   - *Relevant rules*: 2,4
3. Disciplinary researcher wanted data scientist to throw tools at a problem / Disciplinary researcher wanted data scientist to be a service provider
   - *Suggested approach to address them*: Set clear expectations, set each other up for success
   - *Relevant rules*: 2
4. Disciplinary researcher underestimates time required for data scientist
   - *Suggested approach to address them*: In SOP, create timeline
   - *Relevant rules*: 2
5. Disciplinary researcher thinks data scientist can fix everything; "service provider"
   - *Suggested approach to address them*: Establish clear roles and lay out methods that are needed to answer questions
   - *Relevant rules*: 2,4
6. Disciplinary researcher keeps changing the goal during the course of research
   - *Suggested approach to address them*: Reflect on successes and failures and use that to push research forward
   - *Relevant rules*: 5,10
7. Project stalls; unclear vision of next step or project ownership
   - *Suggested approach to address them*: Refer back to SOP, make sure there is a leader appointed
   - *Relevant rules*: 2

**Challenges pertaining to data structure, storage & access**:

1. Disciplinary researcher wanted data and analyses in one location
   - *Suggested approach to address them*: Use a shared platform
   - *Relevant rules*: 6
2. Data scientist was given data in a non-standard format
   - *Suggested approach to address them*: Come up with or agree on a data format and make sure everyone has been on-boarded
   - *Relevant rules*: 3,7
3. Data scientist having a clear understanding of the data and metadata
   - *Suggested approach to address them*: Annotate, Annotate, Annotate. Create a README file. Create a lab notebook of procedures and data collection standards for your field.
   - *Relevant rules*: 7
4. Data scientist did not have access to data needed for analyses

- *Suggested approach to address them*: Create clear rules for data access (anonymize, provide in chunks, etc.)
- *Relevant rules*: 9

5. Data is not licensed: no license is the same as the most restrictive license
    - *Suggested approach to address them*: Decide on data licensing at the start of the project; only use data available for reuse
    - *Relevant rules*: 7

**Challenges pertaining to communication**:

1. Data scientist has a difficult time to identify domain jargon
    - *Suggested approach to address them*: Lay out terms that are integral to your field. Create a lab notebook of key concepts.
    - *Relevant rules*: 3
2. Disciplinary researcher has no idea what data scientist is talking about (statistical power, unsupervised learning, convolutional neural network, ...).
    - *Suggested approach to address them*: Lay out terms that are integral to your field. Create flow charts showing how software and infrastructure relate to the project.
    - *Relevant rules*: 3
3. Domain (data) scientist does not get data (domain) scientist way of thinking.
    - *Suggested approach to address them*: By participating in each other's fields, minds will be opened to new possibilities.
    - *Relevant rules*: 1,4

**Challenges pertaining to knowledge exchange**:

1. Hard to find data scientist with right skill set
    - *Suggested approach to address them*: Attend places with DS groups. Learn the wide range of DS fields.
    - *Relevant rules*: 1
2. Data scientist needs to learn domain's field
    - *Suggested approach to address them*: Attend places with DS groups. Learn the wide range of DS fields. Building a common language can also help with understanding.
    - *Relevant rules*: 1,3
